# Training to achieve coordination of rescue and ambulance and medical teams

**DOI:** 10.1186/cc13259

**Published:** 2014-03-17

**Authors:** T Yamashita, K Yamazumi, H Takayama, Y Sakamoto

**Affiliations:** 1 Saga University, Saga, Japan; 2 Ureshino Medical Center, Ureshino, Saga, Japan; 3 Nagasaki Medical Center, Omura, Nagasaki, Japan

## Introduction

Japanese emergency medical technicians are not allowed to perform some advanced medical practices such as a tracheal intubation, establishment of an intravenous line, administration of a drug to a patient who retains their own circulation. So medical staff have to be dispatched to the accident scene to give a patient such medical practices. In Japan, we have developed and dispersed the educational course of the Disaster Medical Assistance Team since 2005. But the role of medical teams at the scene is not widely known among rescue workers. It is also distant for medical staff to coordinate rescue teams, because most of them do not work with rescue workers in their daily work. We have felt a need for practical training to achieve the coordination of rescue and medical teams.

## Methods

We held a disaster-relief training workshop from 2010 to 2013. Prior to starting the drill, we gave two lectures about the role of a medical team in a rescue site and the basic knowledge of rescue skills. The drill was organized for 1 to 1.5 hours. The scenario was secret for participants. Before and after the workshop, we had a questionnaire survey for attitude changes about a collaborative work between a rescue team and a medical team.

## Results

A total of 160 people participated in the workshop (63 rescue workers, 27 ambulance workers, 33 paramedics, 37 medical staff). At first, rescue workers tended to downplay the importance about the division of roles or the establishment of command and control system between rescue teams and medical teams, but their attitude changed after the drill (*P *< 0.05). They also understood the need to know about basic trauma survey skills and some medical terms.

## Conclusion

To achieve mutual understanding, it is important to have drills in which both rescue teams and medical teams participate.

